# Comparison of Oxidative Effects of Two Different Administration Form of Oxybutynin in the Potential Target Tissues

**DOI:** 10.1155/2018/8124325

**Published:** 2018-12-24

**Authors:** Kaan Kaltalioglu, Fatmanur Tugcu-Demiroz, Fusun Acarturk, Barbaros Balabanli, Sule Coskun-Cevher

**Affiliations:** ^1^Espiye Vocational School, Giresun University, 28600 Giresun, Turkey; ^2^Pharmaceutical Technology Department, Pharmacy Faculty, Gazi University, 06330 Ankara, Turkey; ^3^Biology Department, Science Faculty, Gazi University, 06500 Ankara, Turkey

## Abstract

Oxybutynin is an important anticholinergic agent that prevents uncontrolled contractions in the treatment of overactive bladder (OAB). However, drugs containing oxybutynin have significant side effects such as dry eyes, dry mouth, increased heart rate, constipation, blurred vision, and confusion. In recent years, new delivery methods for this agent are being searched. One of them is vaginal delivery. In this study, we aimed to compare the effects of oxybutynin on oxidative parameters in the potential target tissues of the oral and vaginal delivery. Female New Zealand white rabbits (*n*=12) were divided into two groups: oral delivery and vaginal delivery. The animals were sacrificed 48 h after administration and nitric oxide (NOx), thiobarbituric acid-reactive substances (TBARs), and glutathione (GSH) levels were determined spectrophotometrically in the aorta, salivary gland, and small intestine tissue samples. Vaginal delivery significantly decreased NOx levels in all tissue samples as compared to oral delivery (*p* < 0.05). Moreover, it reduced TBARs levels in salivary gland and aorta tissue samples (*p* < 0.05). In the light on these findings, it can be said that vaginal delivery may decrease the oxidant-induced side effects of oxybutynin as compared to oral delivery.

## 1. Introduction

Overactive bladder (OAB) is a syndrome that occurs owing to the spontaneous activity of the detrusor muscle during the filling of bladder and marked by urinary urgency, with/without urge incontinence, usually accompanied by nocturia and frequency [1, 2]. OAB is common in both men and women, and negatively affects the quality of life (QOL) [3]. Recent reports showed that the prevalence of OAB is 35.15% in United States [4], and 20% in Asia (men and women aged ≥40 years) [5]. Two main classes of drugs have been used for the treatment of OAB: antimuscarinics (anticholinergics) and *β*3-adrenoreceptor agonists [6].

The detrusor muscle contractility is generally orchestrated by the parasympathetic nervous system, which acetylcholine (ACh) is the primary neurotransmitter in these actions [7]. Antimuscarinics inhibits the binding of acetylcholine at M_2_ and M_3_ muscarinic receptors on bladder smooth muscle to show their effects on OAB. Antimuscarinics has demonstrated efficacy for the treatment of OAB, but they affect the potential target organs or tissues, and they can cause side effects for some people [7]. These side effects such as dry mouth, dry eyes, increased heart rate, constipation, confusion, and blurred vision are related to dose [8].

Oxybutynin, a tertiary amine, is an antimuscarinic agent used widely in the treatment of OAB for many years [6]. Oxybutynin has an antimuscarinic and a direct muscle relaxant effect, as well as local anesthetic actions [9]. Nevertheless, it can cause side effects and is discontinued in over 60% of patients due to adverse effects [10]. Oxybutynin have major side effects include dry eyes, dry mouth, blurred vision, and constipation [2]. Oxybutynin is highly lipophilic molecule to have the ability of penetrate the blood-brain barrier [6]. *N*-desethyloxybutynin, which is the major metabolite of oxybutynin, may cause systemic side effects. Oxybutynin and *N*-desethyloxybutynin have similar impacts on the detrusor smooth muscle. But, *N*-desethyloxybutynin is more vigorous in the salivary glands (leads to dry mouth) [11].

Vaginal drug delivery systems promise remarkable potential in pharmacy. The major advantages of this route include a considerable surface area, good blood supply, relatively high permeability to many drugs, and the ability to bypass first-pass liver metabolism [2]. Previous works suggested that the vaginal delivery of oxybutynin may have serious advantages over the oral delivery with relation to long duration of action, consistent blood levels, and reduction of side effects [2].

The imbalance between oxidants and antioxidants (called as oxidative stress) is involved in the etiology and/or pathogenesis of a number of disorders [12]. Reactive oxygen species (ROS) and reactive nitrogen species (RNS) generate a harmful process that can alter the cell membranes and other structures such as proteins, lipids, proteins, and DNA and trigger various diseases [13, 14]. For instance, Sjögren's Syndrome (SS) is an autoimmune disorder, which leads to dry eye and dry mouth. It has been proposed that oxidative stress plays a role in the pathogenesis of SS [15]. It has also been reported that oxidative stress may cause changes in the structure of the salivary gland and may involve in hyposalivation [16]. In addition, nitric oxide may also affect saliva secretion [17].

In this study, we compared the effects of oral and vaginal delivery of oxybutynin on some oxidative parameters (NOx, TBARs, and GSH) on the aorta, salivary gland, and small intestine, organs where oxybutynin may cause side effects.

## 2. Material and Methods

### 2.1. Drugs and Chemicals

In this study, oxybutynin hydrochloride was obtained from Sigma (USA). Also, Üropan® (Koçak Farma, Turkey) tablet (5 mg oxybutynin) was purchased from markets, and all other chemicals unless until mentioned were of analytical grade.

### 2.2. Experiment

The protocol of this study was confirmed by the Animal Ethical Committee of Gazi University, Turkey (G.Ü.ET-04.033). Twelve female New Zealand white rabbits weighing 2.5–3.5 kg were housed them in a controlled environment. During the experimental period animals were allowed free access to food and water. They fasted for 24 h before application. The animals were divided randomly into two groups: oral delivery (*n*=6) and vaginal delivery (*n*=6). Vaginal solution (containing 10 mg oxybutynin/1.5 mL pH 4.5 phosphate buffer) was administered through the vaginal route with an applicator as a single dose every 48 h. Üropan® tablets (5 mg oxybutynin) were also administered orally through an oral gavage as a single dose every 24 h. After 48 h from administration, the animals were killed with intracardiac blood aspiration under ketamine/xylazine anesthesia (intramuscularly, 75 and 5 mg/kg, respectively). The aorta, salivary gland, and small intestine tissue samples were collected, frozen and kept in –80° until assay.

### 2.3. Determination of NOx, TBARs, and GSH

The reactive nitrogen oxide species (NOx) levels were measured spectrophotometrically at 540 nm by Griess reaction. NOx are stable end products of nitric oxide. Sodium nitrite was considered as the standard [18]. Lipid peroxidation was determined spectrophotometrically according to the method detailed before at 535 nm. This method based on the principle that the generation of malondialdehyde (MDA) as an end product of lipid peroxidation, which interacts with thiobarbituric acid producing thiobarbituric acid-reactive substance (TBARs). Tetraethoxypropane solution was considered as a standard for this assay [18]. Levels of glutathione (GSH) were measured spectrophotometrically according to a modified version of the Ellman method at 412 nm. In this method, 5,5′-dithiobis(2-nitrobenzoic acid) (DTNB) reacts with reduced GSH to form a yellow-colored compound [18].

### 2.4. Statistical Analysis

All results were defined as the mean ± standard deviation. One-way ANOVA (with posthoc Tukey test) was used to compare mean differences. Results of *P* < 0.05 were considered to be statistically significant.

## 3. Results and Discussion

**Figure 1:**
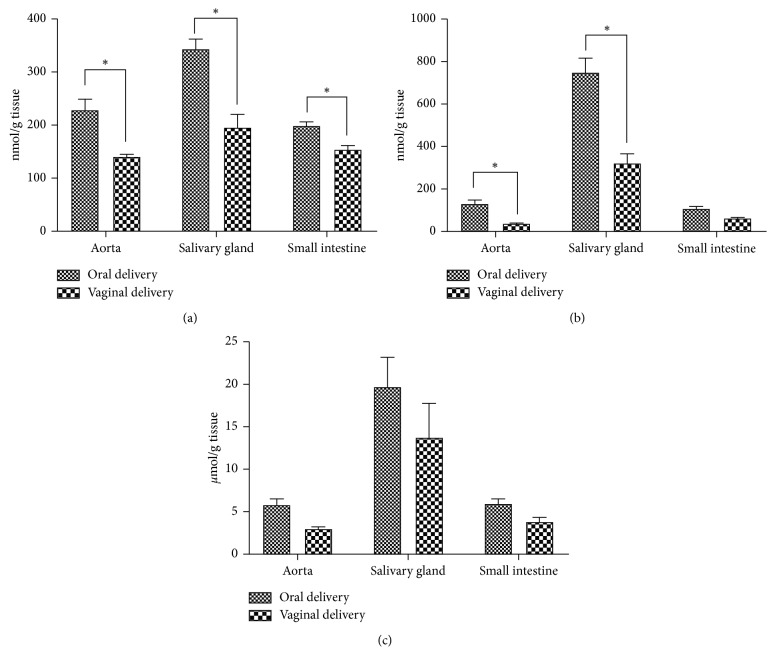
Effects of the oral and vaginal delivery of oxybutynin on NOx (a), TBARs (b), and GSH (c) levels in various tissues. *p* < 0.05 compared to the oral and vaginal delivery group.

The amounts of the NOx, TBARs, and GSH in the aorta, salivary gland, and small intestine tissue samples are presented in Figure 1. Our results showed that in the vaginal delivery groups, the levels of NOx are significantly lower in all tissue samples (aorta (140.54 ± 4.63 nmol/g tissue), salivary gland (195.99 ± 24.03 nmol/g tissue), and small intestine (154.29 ± 7.37 nmol/g tissue)) as compared to oral delivery groups (228.63 ± 20.13 nmol/g tissue, 343.42 ± 18.33 nmol/g tissue, 198.69 ± 7.23 nmol/g tissue, respectively) (*p* < 0.05) (Figure 1). Nitric oxide, a highly reactive gas molecule, has multiple physiological roles in both intra- and extracellular signal transduction pathways with relations for health and disease [19]. The concentration of NO is critical since it acts as a signaling molecule at low concentrations, although, at high concentrations, NO or RNS can exert toxic effects on the cell; it is damage to proteins, lipids, and DNA and can lead to cell death or tissue injury [14, 20]. Salivation is dependent on cholinergic stimulation via autonomic nerves and intracellular signals in acinar cells [21]. NO and acetylcholine (ACh) function in salivation. NO can stimulate salivary gland cells to secrete fluid, but a prolonged exposure has a detrimental effect on the cells [22]. Konttinen et al. reported that NO is an important factor in altering salivation in SS, which is characterized by dry mouth and dry eye. Nitrite is present at high concentrations in saliva of these patients, and inducible NO synthase (iNOS) expression is elevated in various cells in salivary glands [23]. Also, dry mouth is an important side effect of the administration of oxybutynin. Therefore, the preference for vaginal delivery may be beneficial for salivation as it reduces NO concentration.

Otherwise, another side effect of oxybutynin is increased heart rate. NO plays an important role in heart contraction response, heart rate, and regulation of vascular tone [24]. The effects of NO on the heart rate are complex. But, in general, NO has a positive chronotropic effect, and NOS inhibition causes a reduction of heart rate [25]. The different effects of NO might be dependent on its concentration. As shown in Figure 1, the level of NOx in the aorta tissue samples of the vaginal delivery group was significantly lower than oral delivery group (*p* < 0.05).

Constipation is a common side effect that may potentially appear with the use of oxybutynin. Another important function of NO is that it affects the gastrointestinal tract (via relaxation of the enteric smooth muscle). A previous study suggested that NO supports maintain gastric mucosal integrity and has a protective effect [20]. In contrast, in cirrhotic rats, L-NAME (a nonselective inhibitor of NOS) alleviate inhibited-gastrointestinal motility. Wang et al. reported that NO may play an important role in the inhibition of gastrointestinal motility [26]. In another study, Tomita et al. found that NO has a significant function in the dysmotility detected in the STC patientsʼ colons [27]. Taken together, the above results suggest that instead of oral delivery, vaginal delivery may be beneficial in attenuating deleterious effects of NO by reducing the concentration of NO.

A significant decrease in TBARs levels of aorta (36.86 ± 3.88 nmol/g tissue) and salivary gland tissues (322.72 ± 43.17 nmol/g tissue) was observed in the vaginal delivery groups when compared with oral delivery groups (131.41 ± 16.66 nmol/g tissue, 749.15 ± 67.50 nmol/g tissue, respectively) (*p* < 0.05) (Figure 1). Lipid peroxidation products are markers for oxidative stress status *in vivo* and its related diseases. Malondialdehyde (MDA) is an important oxidation product, which is considered as the main marker in lipid peroxidation, and can be measured via TBARs assay [28–30]. Some metabolites are formed by the metabolism of oxybutynin by the cytochrome P450 enzyme system. One of these, *N*-desethyloxybutynin, is a stable and toxic metabolite [31]. This oxybutynin derived toxic metabolite can alter oxidative stability. Oxidative stress may lead to dysfunction of salivary gland [32]. Several studies have shown oxidative stress effects secretion and components (calcium, protein, etc.) of saliva [33, 34]. Additionally, it has been suggested that ROS concerned with the onset and pathology of SS [15, 34, 35]. Similarly to salivation, heart failure may also be associated with oxidative stress. Studies of the causes responsible for the progression of heart failure have shown that reactive oxygen species (ROS) play an important pathological role [36]. Overall, these findings indicate that vaginal delivery may be beneficial as it reduces TBARS levels and hence oxidative stress.

In all tissue samples, the level of GSH in the oral delivery group (aorta (5.83 ± 0.67 *µ*mol/g tissue), salivary gland (19.72 ± 3.45 *µ*mol/g tissue), and small intestine (5.95 ± 0.56 *µ*mol/g tissue)) was found to be higher than vaginal delivery group (2.98 ± 0.23 *µ*mol/g tissue, 13.77 ± 3.97 *µ*mol/g tissue, 3.86 ± 0.48 *µ*mol/g tissue, respectively), but it was not statistically significant (*p* > 0.05) (Figure 1). GSH is a tripeptide acts as a free radical scavenger and is an important member of antioxidant defense system. The reason for this is not clear, but a possible explanation may be that the organism may have increased the level of GSH in order to strengthen the antioxidant defense system in response to increasing oxidative stress (parallel with TBARs result).

## 4. Conclusion

Oxidants, especially nitric oxide, play an important role in the pathophysiology of many diseases. Instead of orally administration, vaginal administration of oxybutynin can alleviate oxidant-induced side effects. These results can contribute to pharmaceutical studies.

## Data Availability

The data used to support the findings of this study are available from the corresponding author upon request.
